# Tolerogenic Function of Dimeric Forms of HLA-G Recombinant Proteins: A Comparative Study *In Vivo*

**DOI:** 10.1371/journal.pone.0021011

**Published:** 2011-07-14

**Authors:** Benoit Favier, Kiave-Yune HoWangYin, Juan Wu, Julien Caumartin, Marina Daouya, Anatolij Horuzsko, Edgardo D. Carosella, Joel LeMaoult

**Affiliations:** 1 CEA-I2BM-Service de Recherches en Hemato-Immunologie, Paris, France; 2 Institut Universitaire d'Hematologie, Hopital Saint Louis, Paris, France; 3 Biology and Biotechnology Ph.D. Program, University Paris 7, Paris, France; 4 Center for Molecular Chaperone/Radiobiology and Cancer Virology, Georgia Health Science University, Augusta, Georgia, United States of America; Centre de Recherche Public de la Santé (CRP-Santé), Luxembourg

## Abstract

HLA-G is a natural tolerogenic molecule involved in the best example of tolerance to foreign tissues there is: the maternal-fetal tolerance. The further involvement of HLA-G in the tolerance of allogeneic transplants has also been demonstrated and some of its mechanisms of action have been elucidated. For these reasons, therapeutic HLA-G molecules for tolerance induction in transplantation are actively investigated. In the present study, we studied the tolerogenic functions of three different HLA-G recombinant proteins: HLA-G heavy chain fused to β2-microglobulin (B2M), HLA-G heavy chain fused to B2M and to the Fc portion of an immunoglobulin, and HLA-G alpha-1 domain either fused to the Fc part of an immunoglobulin or as a synthetic peptide. Our results demonstrate the tolerogenic function of B2M-HLA-G fusion proteins, and especially of B2M-HLA-G5, which were capable of significantly delaying allogeneic skin graft rejection in a murine *in vivo* transplantation model. The results from our studies suggest that HLA-G recombinant proteins are relevant candidates for tolerance induction in human transplantation.

## Introduction

Since the first successful kidney allo-transplantation in human beings in 1952, the development of treatments limiting acute allograft rejection has been the purpose of intense investigations. Even though the discovery of immunosuppressive molecules such as Cyclosporin A dramatically reduced acute allograft rejection cases, their action on chronic allograft rejection is not optimal. Moreover, besides their lack of efficiency on chronic allograft rejection, these immunosuppressive treatments have side effects including high susceptibility to infections, and renal and neural toxicity.

Among the biological molecules involved in the induction of tolerance that have been characterized over the past years, the non-classical HLA class I Human Leukocyte Antigen G molecule (HLA-G) has unique features that make it an ideal candidate for the development of new therapies in transplantation.

HLA-G (reviewed in [1], [2]) is characterized by seven isoforms which derive from the alternative splicing of a unique primary transcript, by a very low amount of polymorphism, and by an expression which is restricted to fetal trophoblast cells, adult epithelial thymic cells, cornea, erythroid and endothelial cell precursors, and pancreatic islets. HLA-G may also be pathologically expressed by (i) non-rejected allografts [3], [4], (ii) lesion-infiltrating antigen presenting cells (APC) during inflammatory diseases [5], [6], and (iii) tumor tissues and their tumor infiltrating APC [7]–[11]. HLA-G is further expressed by (iv) monocytes in multiple sclerosis [12], and by (v) monocytes and T cells in viral infections [13]–[15].

HLA-G is a potent tolerogenic molecule that strongly inhibits the function of immune cells. Indeed, HLA-G inhibits NK cell and cytotoxic T lymphocyte cytolytic activity [16], [17], CD4^+^ T cell alloproliferative responses [18], T cell and NK cell ongoing proliferation [18]–[20], and dendritic cell maturation [21], [22]. Furthermore, HLA-G was shown to induce regulatory T cells [18], [23]. HLA-G mediates its functions by interacting with three inhibitory receptors: ILT2 (CD85j/LILRB1) which is expressed by B cells, some T cells, some NK cells and all monocytes/dendritic cells [24], ILT4 (CD85d/LILRB2) which is expressed by myeloid cells [25], and KIR2DL4 (CD158d) [26] which is expressed by some peripheral and decidual NK cells.

The efficiency of the HLA-G binding to its receptors and the delivery of potent inhibitory signals have been shown to depend on HLA-G dimerization [27]. Biochemical studies indicate that HLA-G dimerization occurs through disulfide-bond formation between unique cysteine residues localized in position 42 of the HLA-G alpha-1 domain (C42). Point mutation of C42 in Serine, which leads to the exclusive expression of HLA-G monomers demonstrated that HLA-G dimers, but not HLA-G monomers, carry HLA-G tolerogenic function [27], [28].

The expression of HLA-G dimers has been reported in trophoblast cells, where it confers protection against the mother's immune system. This mechanism of natural tolerance in a semi-allogeneic context has led to investigate the potential role of HLA-G in transplanted patients (reviewed in [2]). To date, clinical studies have demonstrated that HLA-G expression may be induced in some heart, kidney, liver/kidney, lung, pancreas, and kidney/pancreas transplanted patients. Statistical analyses indicate that the presence of HLA-G in plasma and biopsies of transplanted patients correlates with a decreased number of acute rejection episodes and with no chronic rejection, as first described for heart transplants [3], [29].

The direct role of HLA-G in transplantation *in vivo* was evidenced by skin allotransplantation in HLA-G transgenic mice or in wild-type mice pre-treated with HLA-G tetramer-coated beads. In both experiments the presence of HLA-G significantly delayed skin allograft rejection [30], [31].

For these reasons, and also because it already contributes to the best example of successful tolerance there is: the maternal-fetal tolerance, therapeutic HLA-G molecules for transplantation are actively investigated. Yet, the use of HLA-G molecules as therapeutic agents faces several hurdles, among which the problems of structure and stability. Indeed, HLA-G is a trimolecular complex composed of a heavy chain of 3 globular domains non-covalently associated with the β2-microglobulin (B2M) and a peptide which is active only as a multimer.

Here, we evaluated (i) the tolerogenic function of two types of HLA-G homodimers (C42-C42 dimers *vs* Fc-Fc dimers), (ii) whether the alpha-1 domain of HLA-G which is common to all HLA-G isoforms could carry a tolerogenic function by itself as it was originally postulated, and (iii) whether the trimolecular complex that constitutes HLA-G could be stabilized by fusing B2M to HLA-G heavy chain while retaining its tolerogenic properties.

Our results demonstrate the tolerogenic function of all investigated dimeric forms of HLA-G recombinant proteins *in vitro* and *in vivo*, and especially that of the B2M-HLA-G5 dimers *in vivo*, but do not fully support a tolerogenic function for the alpha-1 domain of HLA-G in human beings, even dimeric.

## Materials and Methods

### Engineering of HLA-G fusion proteins

#### B2M-HLA-G1s-Fc (β2-microglobulin fused to the extracellular part of HLA-G1 and to the Fc part of a human IgG)

The sequence coding for the human β2-microglobulin (B2M) was amplified by PCR with the primers B2M_Sig_Mlu-I_Sph-I_F and B2M-Link-a1_R (Table 1). The B2M_Link-a1_R primer, which is reverse complementary to the B2M coding sequence, is constituted of three parts: B2M 3′ sequence excluding the stop codon, fused to a sequence coding for a (GGGGS)×2 linker, fused to the reverse complementary sequence of the HLA-G alpha-1 domain 5′ end.

**Table 1:** Primers used for fusion protein generation.

B2M_Sig_Mlu-I_Sph-I_F	5′ CGTCGCATGCACGCGTCGATGTCTCGCTCCGTGGCC 3′
B2M-Link-a1_R	5′ TCATGGAGTGGGAGCCGGATCCGCCACCTCCGGATCCGCCACCTCCGGATCCGCCACCTCCCATGTCTCGATCCCACTT 3′
HLA-G-a1_Mlu_Sph_F	5′ ACTGGCATGCA*C*GCGTCGGGCTTCCACTCCATGA 3′
HLA-G-a3_Xho_Sal_R	5′ TATGGTCGACCTCGAGCGCAGCTGCCTTCCATCTCAGCATGAG 3′
B2M_sig_TOPO_F	5′ CACCATGTCTCGCTCCGTGGCC 3′
a3_i4_Xho_Stop_R	5′ ATCTTAACTCGAGAGGTCTTCAGAGAGGCTCCTGCTTTCC>TAACAGACATGATGCCTCCATCTCCCTCCTTACTCCATCTCAGCATGAG 3′
EcoRI_a1_F	5′ AAAGAATTCGGGCTCCCACTCCATGAGGT 3′
EcoRV_a1_R	5′ AAAGATATCCCACTGGCCTCGCTCTGGTTG 3′

In parallel, the cDNA sequence corresponding to the α1 through α3 domains of HLA-G1 was amplified by PCR with primers HLA-G-a1_Mlu_Sph_F and HLA-G-a3_Xho_Sal_R (Table 1). This removed the HLA-G peptide leader sequence and the stop codon. The PCR fragments corresponding to B2M and the α1-α2-α3 domains of HLA-G were then digested with the Eag-I restriction enzyme, purified, and ligated together. Mlu-I and Xho-I were then used to insert the obtained B2M_α1-α2-α3 fusion sequence (B2M-HLA-G1s) into a PGEMT/easy vector (Promega). The restriction enzymes Age-I and Xho-I were used to transfer the B2M-HLA-G1s sequence to the pFUSE-hFc1 (InVivogen, Toulouse, France) in order to fuse B2M-HLA-G1s to the Fc part of a human IgG.

#### B2M-HLA-G5 (β2-microglobulin fused to the HLA-G5 heavy chain)

To generate B2M fused to the heavy chain of the HLA-G5 isoform, the pFUSE-B2M-HLA-G1s-Fc plasmid described above was used as template and amplified with B2M_sig_TOPO_F and a3_i4_Xho_Stop_R (Table 1). a3_i4_Xho_Stop_R contains the HLA-G intron 4 sequence that replaces the transmembrane and intracellular and domains of HLA-G1 in HLA-G5. The PCR fragment was then ligated into the pcDNA 3.1 D/V.5-His-Topo vector (Invitrogen) using 3.1 Directional TOPO® Expression Kit (Invitrogen).

#### Alpha1-Fc (HLA-G alpha-1 domain fused to the Fc part of a murine IgG)

The cDNA sequence of HLA-G alpha-1 domain was amplified by PCR using the primers EcoRI_a1_F and EcoRV_a1_R (Table 1). The EcoRI and EcoRV restriction enzymes were used to insert the HLA-G alpha-1 coding sequence into the pFUSE-mFc2 vector (Invivogen), fusing it to the secretion sequence of IL-2 and a mouse IgG Fc fragment.

#### Alpha1_peptide (synthetic peptide corresponding to the HLA-G alpha-1 domain)

The peptide corresponding to the alpha-1 domain of HLA-G was synthesized by Jerini, Berlin, Germany.

**Figure 1:**
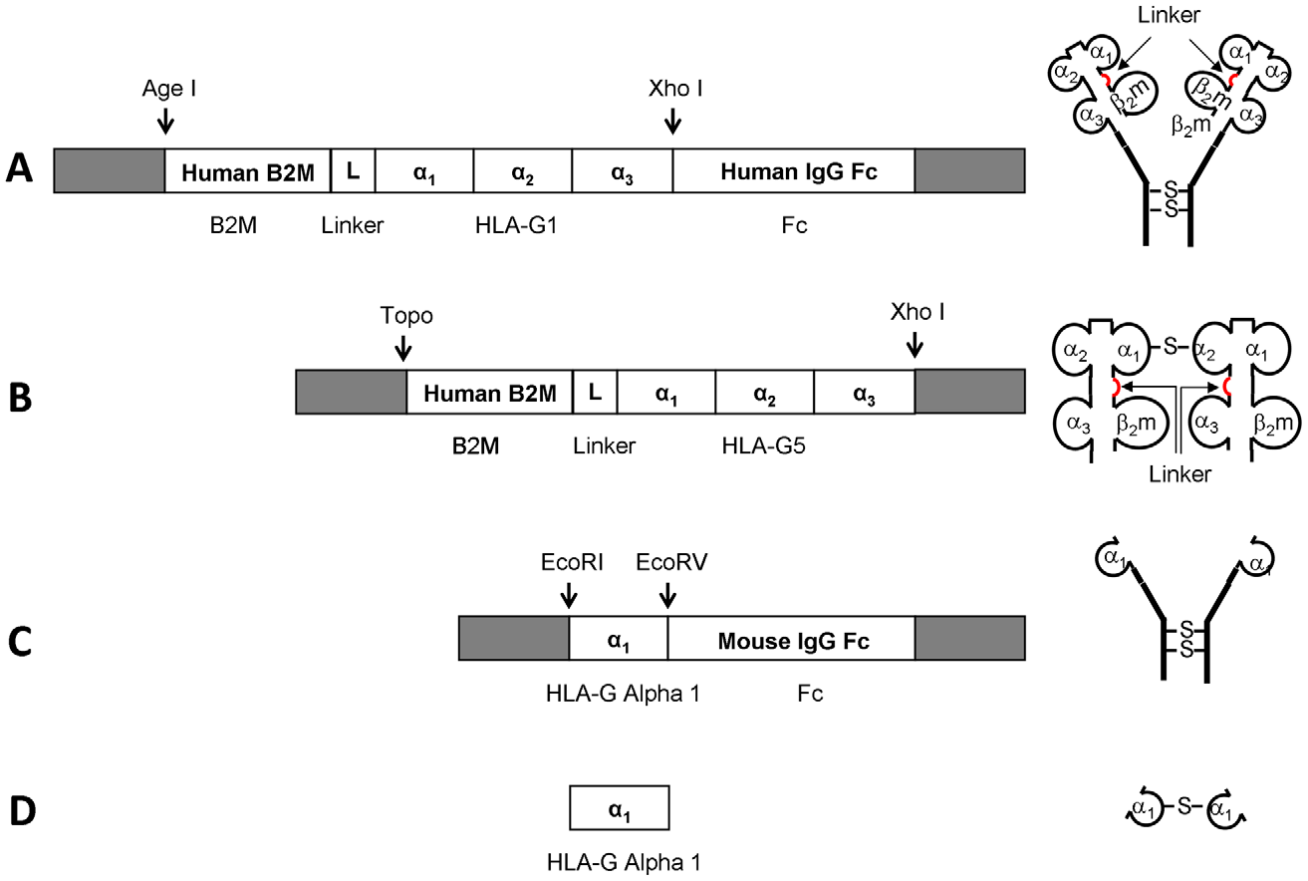
Schematic representation of the generated proteins and their coding sequences. (A) B2M-HLA-G1s-Fc, (B) B2M-HLA-G5, (C) Alpha1-Fc, (D) Alpha1_peptide. Arrows indicate the linker between beta-2-microglobulin and HLA-G alpha-1 domain.

Figure 1 is a schematic representation of the generated proteins and peptides.

### Cell culture and proteins production

For production of HLA-G fusion proteins, the HeLa human cell line (ATCC) was transfected by the various plasmids using the Lipofectamine™ 2000 transfection reagent (Invitrogen) and kept at 37°C, 5% CO_2_ in DMEM (Dulbco's Modified EagleMedium GIBCO) GlutaMAX™ supplemented with 10% fetal calf serum (GIBCO) and the appropriate selective antibiotics. For B2M-HLA-G1s-Fc and Alpha1-Fc, zeocin™ was used at a concentration of 500 µg/mL. For B2M-HLA-G5, geneticin was used at a concentration of 1 mg/mL.

For the collection of fusion protein-containing culture supernatants, cells were harvested after 48 hours. Supernatants were filtered through 0.2 µM filter (Millipore) and then used as is, or stored at −20°C.

### HLA-G-specific ELISA

HLA-G-specific ELISAs were performed on cell culture supernatants using Mem-G/09 (Exbio, Praha) as capture antibody and anti-B2m as detection antibody as described [32]. Purified HLA-G5 was used as standard protein. Samples were run as duplicates and represented as mean+/−SEM.

### Western-blot analysis

#### Immunoprecipitation

HLA-G-Fc proteins were immunoprecipitated from cell culture supernatants using Protein G sepharose beads (GE Healthcare). B2M-HLA-G5 fusion proteins were immunoprecipitated from supernantants using anti-HLA-G5-coated Protein G sepharose beads (clone 5A6, Exbio, Praha). Supernatants of non-transfected cells were used as negative controls. Immunoprecipitates were then washed three times with 1×PBS and the proteins were eluted by incubation in Laemmli buffer under reducing or non-reducing conditions, and boiled before loading.

#### Western blots

Samples were loaded on polyacrylamide SDS-PAGE gels and transferred onto Hybond nitrocellulose membranes (Amersham Pharmacia Biosciences). Following blocking with 5% non-fat milk in TBS/0,2% Tween 20, membranes were incubated overnight with anti-HLA-G antibody (clone 4H84, Exbio, Praha), and revealed using HorseRadish peroxydase-conjugated goat anti-mouse secondary antibody. Membranes were revealed with the ECL^+^ detection system (Amersham Pharmacia Biosciences).

### ILT2 NFAT-GFP reporter cell assay

NFAT-GFP reporter cells expressing the ILT2-PILRβ chimera were described previously [22]. The cell-surface expression of ILT2-PILRβ chimera was monitored by FACS staining with PE-Cy5-conjugated ILT2-specific mAb (clone GHI/75, BD Biosciences). In this system, GFP expression can be induced only upon proper ILT2 receptor binding.

### Mice

C57BL/6 and B6.C-H-2^bm12^ (bm12) mice were purchased from Jackson Laboratory. ILT4-transgenic mice have been described in [21]. The use of animals for this work was approved by the animal care committee of the Medical College of Georgia.

### *In vivo* allograft experiments

The *in vivo* experimental procedures were approved by the animal care committee of the Medical College of Georgia (approval ID: BR08-06-070) and the experiments were conducted in accordance with institutional guidelines for animal care and use.

For B2M-HLA-G1s-Fc and alpha1-Fc fusion proteins, 1×10^8^ sulfate latex beads 4%w/v 5 µm (Invitrogen) were coated with 20 µg/ml AffiniPure anti-human IgG Fc fragment or Goat Anti-mouse, respectively (Jackson ImmunoResearch) for 2 hours at 37°C followed by a 2-hour incubation with BSA (2 mg/ml). After washing, the beads were incubated with 0.5 µg/ml of HLA-G/Fc fusion proteins at 4°C for 16 hours. Subsequently, the beads were washed twice with PBS. 5 ml of HLA-G-Fc fusion proteins (1 µg/ml) were used for 5×10^6^ sulfate latex beads. As a negative control, sulfate latex beads were prepared in an identical manner except that PBS or HeLa mock supernatant was used rather than supernatants containing HLA-G-Fc fusion proteins.

For B2M-HLA-G5, beads were generated by grafting the anti-HLA-G5 antibody 5A6 (Exbio, Praha) on beads prior to incubation in B2M-HLA-G5-containing culture media.

For alpha1_peptide, beads were generated by direct coating onto latex beads.

Sulfate latex beads (5×10^6^) were injected intraperitoneally on the day before skin grafting. Specific pathogen-free C57BL/6 mice and ILT4-transgenic mice (both H-2b, 8–10 weeks of age) were used as skin graft recipients throughout the study. Recipient mice received HLA-G-coupled latex beads, donor skin was from MHC class II-disparate B6.CH-2bm12 (bm12, H-2b) mice. Allogeneic skin grafts were performed by standard methods. Briefly, skin (1.0 cm^2^) from the tail of donor mice (12–14 weeks old) was grafted onto the flank of recipient, anesthetized mice. The graft was covered with gauze and plaster, which were removed on day 10. Grafts were scored daily until rejection (defined as 80% of grafted tissue becoming necrotic and reduced in size). All skin grafting survival data were tested by Kaplan Meier Survival Analysis.

## Results

### Structural validation of the HLA-G recombinant proteins

We first investigated the conformation of the recombinant proteins, focussing primarily on their monomeric/multimeric status, and then on the conformation of the B2M-HLA-G fusion proteins.

**Figure 2:**
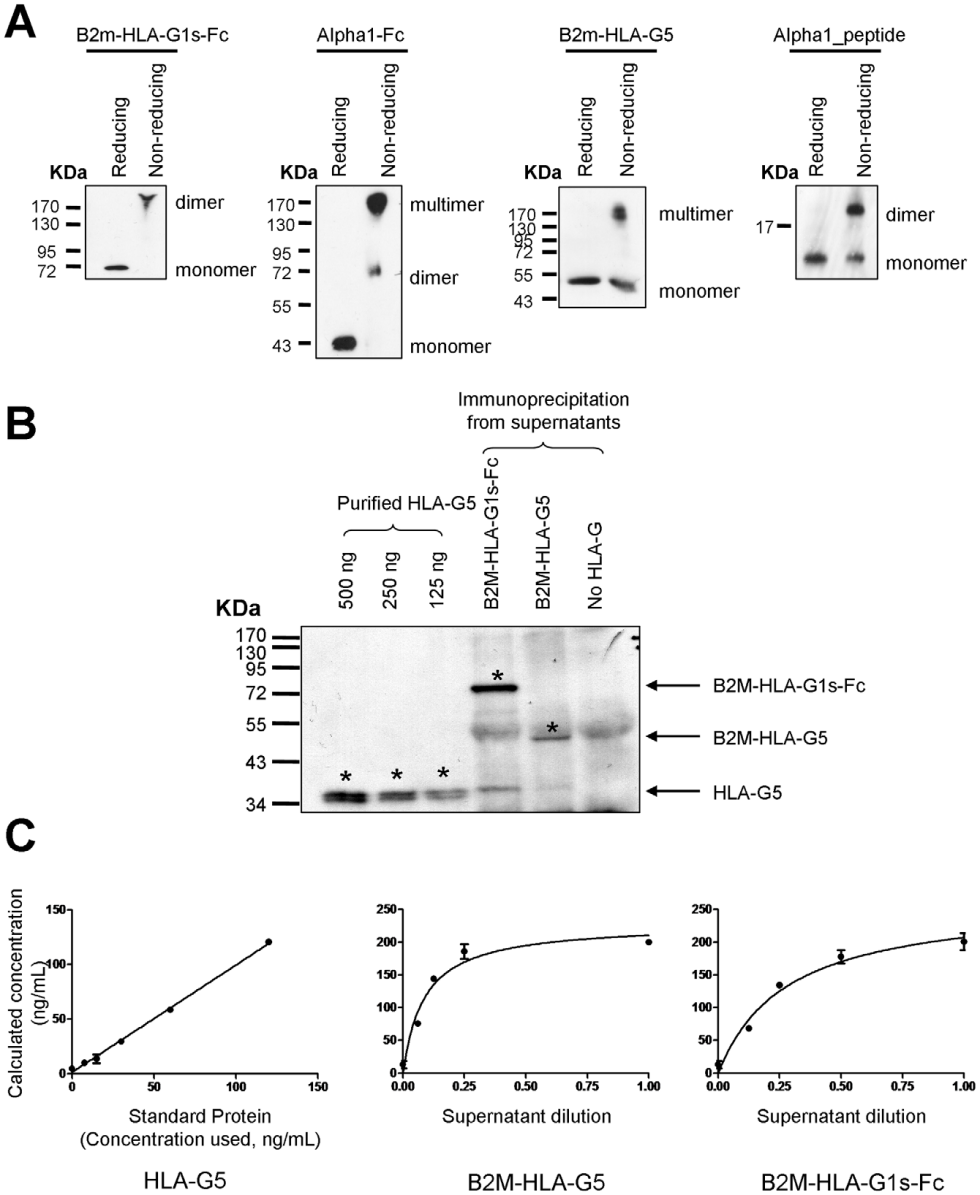
Monomeric/multimeric status and conformation of recombinant proteins. ***A*** *Monomeric/multimeric status of recombinant proteins*. Western blot analysis of recombinant proteins immunoprecipitated from supernatants of HeLa B2M-HLA-G1s-Fc, HeLa Alpha1-Fc, HeLa B2M-HLA-G5, and of alpha1_peptide. ***B*** *Quantification of B2M-HLA-G5 and B2M-HLA-G1s-Fc proteins*. Western blot analysis of recombinant B2M-HLA-G5 and B2M-HLA-G1s-Fc proteins immunoprecipitated from cell culture supernatants. Purified HLA-G5 recombinant protein was used as quantification standard. ***C*** *Conformation of B2M-HLA-G5 and B2M-HLA-G1s-Fc proteins*. Recombinant HLA-G5 protein was used as a standard in HLA-G-specific ELISA. Curves represent the concentration of the proteins properly folded into the supernatant according to the dilution.

To investigate the monomeric/multimeric status of the engineered proteins, these were immuno-precipitated using protein G-Sepharose beads either directly (for Fc-containing proteins), or after binding to anti-HLA-G5 mAb (for B2M-HLAG5). No immunoprecipitation was required for alpha1_peptide. Proteins and peptide were then analyzed by electrophoresis on non-reducing gels. Figure 2A shows that Fc fusion proteins were present only as dimers, and that B2M-HLA-G5 and alpha1_peptide also efficiently formed multimers, even if some monomeric structures remained. Thus, all generated proteins and peptide were capable of forming dimers and/or multimers and found mainly as such.

We next investigated whether the B2M-HLA-G5 and B2M-HLA-G1s-Fc fusion proteins were properly folded. For this, we took advantage of the existence of an ELISA assay based on the conformational anti-HLA-G antibody MEM-G/09 which recognizes only HLA-G folded in combination with B2M. We performed conformational ELISA on supernatants which contained similar amounts of B2M-HLA-G1s-Fc, B2M-HLA-G5, using purified HLA-G5 protein as standard. Figure 2C shows these similar amounts of B2M-HLA-G1s-Fc, B2M-HLA-G5, and HLA-G5 control protein, were detected by conformational ELISA with similar efficiency. This means that the (GSSS)×2 spacer linking B2M and HLA-G heavy chain did not alter the B2M:HLA-G conformation. These results are in agreement with a previous study on a similarly produced B2M-HLA-A2 fusion protein [33]. The absence of conformational antibodies for the alpha-1 domain of HLA-G prevented a similar investigation for the alpha1-Fc construct.

Thus, B2M-HLA-G5, B2M-HLA-G1s-Fc, and alpha1-Fc were dimerized, properly folded (B2M-HLA-G5 and B2M-HLA-G1s-Fc), and thus, potentially capable of being functional.

### *In vitro* binding to ILT2

**Figure 3:**
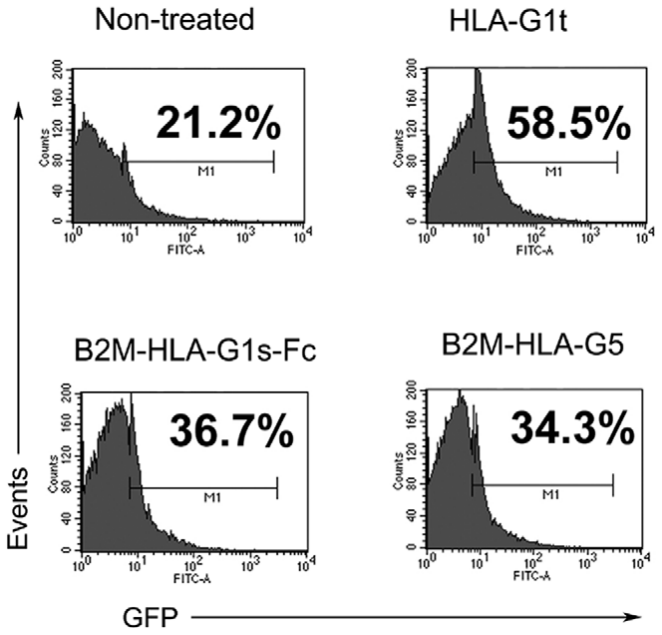
ILT2-mediated signaling by B2M-HLA-G5 and B2M-HLA-G1s-Fc proteins. NFAT-GFP reporter cells expressing the ILT2-PILRβ chimera were stimulated for 16 h with 1.5 µg/ml of the indicated HLA-G recombinant proteins. Non-treated reporter cells were used as negative control, and tetrameric complexes of HLA-G1 (HLA-G1t, [31]) were used as positive control. GFP expression on reporter cells was analyzed by flow cytometry. Numbers indicate the percentage of GFP-positive cells. Data shown are from one of four independent experiments.

*In vitro*, the inhibitory effect of HLA-G is mainly due to its interaction with ILT2, as shown by HLA-G:ILT2 interaction blocking experiments [20]. Furthermore, it was reported that ILT2 binds primarily to dimers of B2M-associated HLA-G molecules, but not to monomers [22], [34]. Thus, we investigated the capability of B2M-HLA-G5 and B2M-HLA-G1s-Fc proteins to bind and activate ILT2. For this, we used an ILT2 NFAT-GFP reporter cell assay in which HLA-G binding to, and activation of ILT2 leads to GFP expression [22]. The results in Figure 3 show that B2M-HLA-G5 and B2M-HLA-G1s-Fc molecules induced GFP expression, i.e. ILT2 activation. Thus, these two molecules had the potential of being functional. Alpha1-Fc and alpha1_peptide were not investigated, since it is known that ILT2 does not bind HLA-G through its alpha-1 but through its alpha-3 domain [35].

### *In vivo* function of recombinant HLA-G molecules

HLA-G promotes skin allograft survival in mice [30]. This has been demonstrated by immunization of wild type and ILT4-transgenic graft recipients mice with recombinant HLA-G 24 hours prior to skin grafting [21], [31]. In these experiments, HLA-G was provided as recombinant soluble HLA-G1 produced in bacteria, refolded with B2M, and then tetramerized prior to be coated onto latex beads. In the current study, HLA-G was provided as B2M-HLA-G5 bound to anti HLA-G5-coated beads (5A6 mAb), B2M-HLA-G1s-Fc bound to anti-Fc-coated beads, alpha1-Fc bound to anti-Fc-coated beads, and alpha1_peptide covalently bound to beads (B2M-HLA-G5 beads, B2M-HLA-G1s-Fc beads, alpha1-Fc beads, and alpha1_peptide beads, respectively). Control beads were mAb-coated beads alone (no HLA-G) or non-coated beads (control for alpha1_peptide beads).

**Figure 4:**
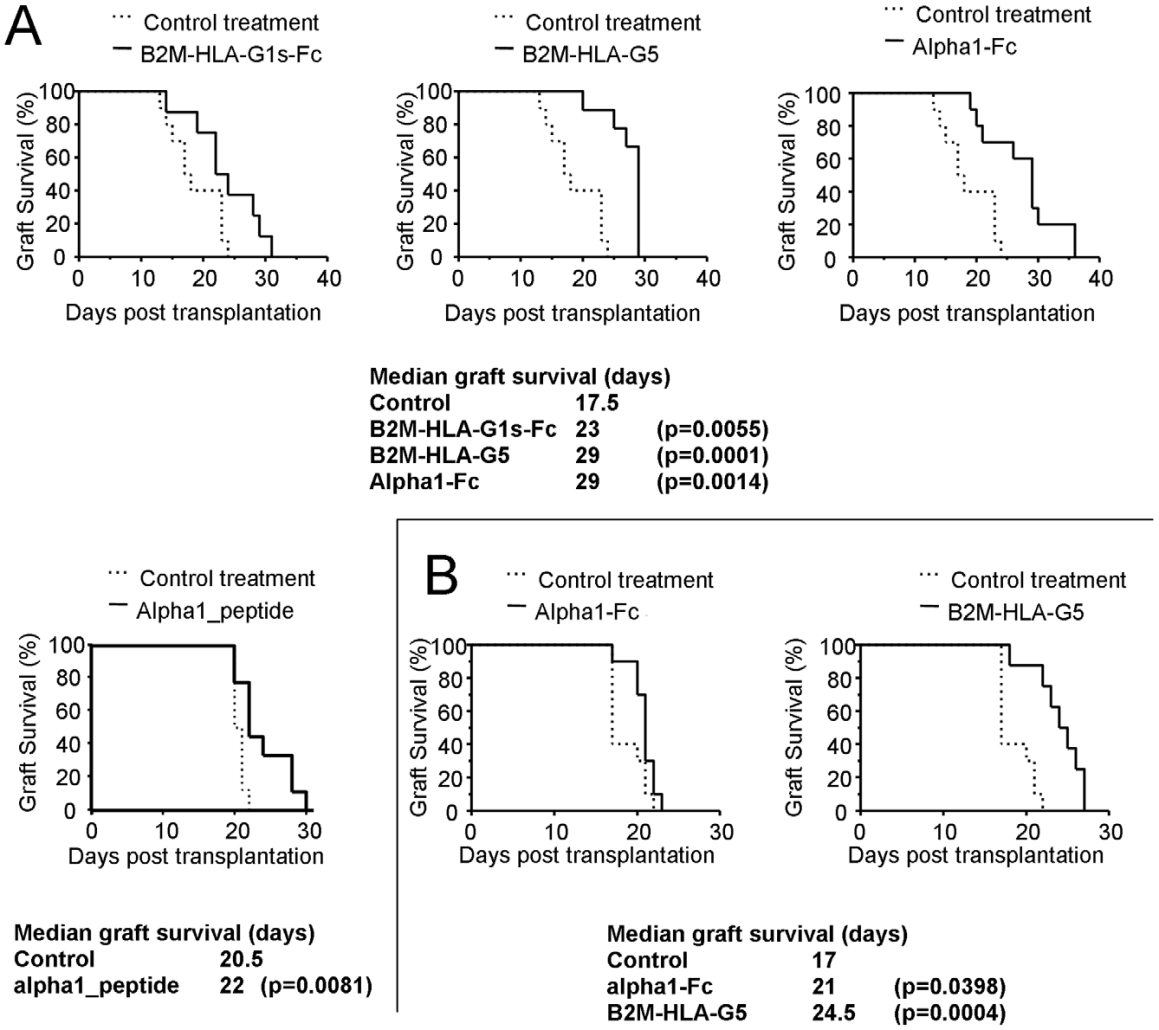
Tolerogenic function of HLA-G fusion proteins and peptide. **A.** C57BL/6 mice strongly recognize the MHC class II-disparate mutant bm12 mouse that carries the I-Abm12 alloantigen. The capability of HLA-G-coated beads to delay rejection was evaluated. Control treatment (dotted lines): beads coated with mAb but without HLA-G proteins. Results are expressed as Median of graft survival time. Kaplan-Meier curves representing graft survival are shown for each HLA-G protein/peptide for controls (plain lines). Associated values are indicated as a table underneath the curves. **B.** The same experiments were performed using ILT4-transgernic mice as skin graft recipients, and for Alpha1-Fc and B2M-HLA-G5. **Tables**: Median survival of transplant, number of animals, and significance are indicated below the corresponding survival graphs.

The results obtained in the case of a MHC class II-disparate B6.C-H-2^bm12^ (bm12) to C57BL/6 allogeneic skin transplantation are shown in Figure 4A. Immunization of the recipient with mAb-coated control beads prior to transplantation had no effect on allograft survival, ruling out any effect of the beads or animal manipulation on the results (not shown). However, all recombinant HLA-G proteins proved to be tolerogenic. B2M-HLA-G5 beads were the most efficient and increased the median graft survival time from 18 to 29 days (n = 9, p = 0.0001). By comparison, B2M-HLAG1s-Fc beads were less efficient but still increased the median graft survival time from 18 to 23 days (n = 8, p = 0.055). Alpha1-Fc beads prolonged skin allograft median survival time, from 18 to 29 days (n = 10, p = 0.001), but by comparison, alpha1_peptide-coated beads were less efficient, increasing the median graft survival from 20.5 to 22 days only (n = 9, p = 0.0081). These results demonstrate an inhibitory effect observed *in vivo* for all fusion proteins and for alpha1_peptide. It is surprising that B2M-HLA-G5 was more efficient than B2M-HLA-G1s-Fc, since these two molecular constructs have the same B2M-HLA-G sequence. It is even more surprising to observe a tolerogenic effect of alpha1-Fc molecules and alpha1_peptide. Indeed, it is known that HLA-G-induced increased survival of skin allograft time is mediated through PIR-B [31], which shares sequence similarities with ILT molecules, and in particular with ILT4. However, ILT4 binds HLA-G through its alpha-3 domain, but does not recognize its alpha-1 domain [36].

Thus, to investigate this issue, we evaluated the tolerogenic function of B2M-HLA-G5 and alpha1-Fc recombinant proteins using ILT4-transgenic mice as skin allograft recipients. B2M-HLA-G5 and Alpha1-Fc were chosen because they were the two most efficient B2M-HLA-G and alpha1 structures.

Figure 4B shows that in this configuration, B2M-HLA-G5 retained its tolerogenic function, increasing the median graft survival time from 17 to 24.5 days (n = 8, p = 0.0004), whereas alpha1-Fc did not (n = 10, p = 0.039).

## Discussion

In this work, we investigated the tolerogenic properties of HLA-G recombinant proteins. These were B2M-HLA-G heavy chain fusions and HLA-G alpha-1 domains dimerized either through an Fc fragment, or naturally through a C42-C42 disulfide bond. Of note, because of the presence of B2M and the linker in our constructs, the cysteines involved in HLA-G homodimerization were no longer in position 42, but for the sake of clarity, we kept calling C42 the cysteines of the HLA-G alpha-1 domain that are responsible for homodimerization.

Our first aim was to evaluate the tolerogenic function of two types of single-chain B2M-HLA-G homodimers. In this study, we showed that all generated proteins and peptide were multimerized. Furthermore, B2M-HLA-G fusions were properly folded and could bind and activate the ILT2 receptor. These data also showed that with respect to binding to and signaling through ILT receptors, dimerization through C42-C42 disulfide bonding or Fc did not seem to matter. This seemed to be confirmed by *in vivo* data, which showed that both dimer types were functional. Yet, B2M-HLA-G5 was more efficient than B2M-HLA-G1s-Fc, and alpha1-Fc was more efficient than alpha1_peptide *in vivo*. It is possible that Fc-dimers and natural dimers might not be structurally identical: whereas dimers formed via C42-C42 bonds are likely to closely resemble “natural” HLA-G dimers, dimers formed via Fc might not. As far as HLA-G structure is concerned, B2M-HLA-G1s-Fc dimers might actually be two HLA-G monomers next to each other rather than “real” HLA-G dimers, although additional dimerization through the C42 residues of HLA-G molecules cannot be ruled out and might happen through the C42 of B2M-HLA-G1s-Fc HLA-G portions located within the same homodimer or not. The same hypothesis can be made for alpha1 constructs: alpha1_peptides may only dimerize through C42-C42 disulfide bridging, whereas alpha1-Fc proteins may multimerize further. Our data seem to indicate that for B2M-HLA-G structures, natural multimers are more efficient than Fc-multimers (B2M-HLA-G structures), and that multimers are more efficient than dimers (alpha1 structures). Whether this will hold true when soluble HLA-G multimers and not bead-bound multimers are used is currently under investigation.

It was reported that all isoforms of HLA-G have immunosuppressive functions [16], including HLA-G3 which extracellular part is constituted of the alpha-1 domain only and which was shown to block the functions of NK cells and CTLs. The other goal of this study was to determine if tolerance induction *in vivo* could be induced by the alpha-1 domain of HLA-G only. For this purpose, alpha1-Fc molecules and a synthetic peptide of HLA-G alpha-1 domain were produced. Alpha1-Fc molecules multimerized, whereas alpha1_peptide molecules dimerized. Interestingly, *in vivo* data showed that the alpha-1 domain of HLA-G prolonged the survival of allo-transplanted skin in mice. This was especially true of alpha1-Fc molecules. Once again, this was unexpected because HLA-G-induced tolerance in mice is mediated through HLA-G binding to PIR-B. This receptor shares sequence similarity with the human ILT family of molecules, and particularly with ILT4 which is known to bind HLA-G alpha-3 domain. One explanation for this could be that when it is not part of the HLA-G1:B2M:peptide complex, the HLA-G alpha-1 domain adopts a conformation that allows it to bind to PIR-B, in which case it might also bind ILT molecules. One other explanation could be that HLA-G alpha-1 domain cross-reacts with inhibitory molecules other than PIR-B, such as murine KIRs for instance. In order to discriminate between these two hypotheses, we tested B2M-HLA-G5 and alpha1-Fc in skin transplantation experiments in which the recipient was an ILT4-transgenic mouse. In these experiments, B2M-HLA-G5 retained its tolerogenic capability, whereas alpha1-Fc almost lost it all. It remains unclear why alpha1-Fc was less tolerogenic in ILT4-transgenic mice than in wild type mice, given that their background was identical, although one can hypothesize that ILT4 may have behaved as a dominant positive PIR-B homologue, thus functionally replacing it. Regardless of the reason, these data clearly show that alpha1-Fc was unable to function through ILT4, whereas B2M-HLA-G5 could. Thus, the originally observed tolerogenic function of alpha1-Fc *in vivo* was most likely due to a specific interaction with PIRB that does not happen with ILT4, or to a cross-reaction with a murine receptor other than PIR-B, indicating that HLA-G alpha1 multimers may function through other receptors than ILT molecules. KIR2DL4, a HLA-G specific receptor which is supposed to bind the alpha-1 domain of HLA-G was not present in our system (no shown) and was also ruled out. Consequently, the mechanism by which HLA-G alpha1 multimers function remains unknown for lack of a receptor, which weakens its position as a candidate tolerogenic molecule to be used in human beings.

Nevertheless, it remains that the alpha-1 domain of HLA-G is present on all HLA-G isoforms. In the light of the data gathered, we propose that one of the main functions of the alpha-1 domain of HLA-G may not be to participate directly in immune inhibition, but to induce dimerization, which is required for ILT binding and for proper function. In this context, it seems that the most stable and active forms of HLA-G might indeed be B2M-associated HLA-G1/-G5, or, as an alternative possibility, HLA-G2/-G6 dimers, containing only the alpha-3 domain required for ILT binding and the alpha-1 domain necessary for dimerization.

In this study, we have demonstrated the tolerogenic properties of artificial single-chain B2M:HLA-G dimers *in vivo* in the context of allogeneic skin transplantation. *In vivo* tolerization was achieved according to a protocol developed with refolded B2M:HLA-G tetramer-coated beads. Apparently, the B2M-HLA-G structures presented here did not perform better than refolded ones. However, it has to be noted that tolerization was obtained by a single injection of HLA-G-coated beads, which is an impressive tolerogenic effect. The advantage of dimerized B2M-HLA-G single-chains over refolded HLA-G tetramers might come from increased stability, which would allow for a longer tolerogenic effect and better prospects for use as soluble molecules rather than coated on beads. The suitability of these constructs for tolerance induction, as well as their *in vivo* stability are currently under investigation.
